# Intermittent scanning continuous glucose monitoring is safe and useful in postsurgical glucose monitoring after pancreatoduodenectomy

**DOI:** 10.1007/s00592-023-02158-0

**Published:** 2023-08-04

**Authors:** Katarina Fagher, Eva Ekström, Jenny Rystedt, Bobby Tingstedt, Bodil Andersson, Magnus Löndahl

**Affiliations:** 1 Department of Clinical Sciences, Lund University, Lund, Sweden; 2 Department of Endocrinology, Skåne University Hospital, 22185 Lund, Sweden; 3 Department of Surgery, Skåne University Hospital, Lund, Sweden

**Keywords:** CGM, Continuous glucose monitoring, Hospital, Surgery, Total parenteral nutrition

## Abstract

**Aims:**

Intermittently scanned continuous glucose monitoring (isCGM) systems have not been thoroughly evaluated during in-hospital stay, and there are concerns about accuracy during various conditions. Patients undergoing pancreatoduodenectomy have an increased risk of hyperglycaemia after surgery which is aggravated by parenteral nutrition therapy. This study aims to evaluate glycaemic control and safety during insulin infusion in a surgical non-ICU ward, using a hybrid glucose monitoring approach with isCMG and periodic point-of-care (POC) testing.

**Methods:**

We prospectively included 100 patients with a resectable pancreatic tumour. After surgery, continuous insulin infusion was initiated when POC glucose was > 7 mmol/l and titrated to maintain glucose between 7 and 10 mmol/l. Glucose was monitored with isCGM together with intermittent POC, every 3–6 h. Median absolute relative difference (MARD) and hypoglycaemic events were evaluated. Mean glucose was compared with a historic control (*n* = 100) treated with multiple subcutaneously insulin injections, monitored with POC only.

**Results:**

The intervention group (isCGM/POC) had significantly lower POC glucose compared with the historic control group (8.8 ± 2.2 vs. 10.4 ± 3.4 mmol/l, *p* < 0.001). MARD was 17.8% (IQR 10.2–26.7). isCGM readings were higher than POC measurements in 91% of the paired cases, and isCGM did not miss any hypoglycaemic event. About 4.5% of all isCGM readings were < 3.9 mmol/l, but only six events were confirmed with POC, and none was < 3.0 mmol/l.

**Conclusions:**

A hybrid approach with isCGM/POC is a safe and effective treatment option in a non-ICU setting after pancreatoduodenectomy.

**Supplementary Information:**

The online version contains supplementary material available at 10.1007/s00592-023-02158-0.

## Introduction

Intermittently scanned continuous glucose monitoring (isCGM) systems are nowadays widely accepted in diabetes care. However, the in-hospital use of these systems has yet to be fully evaluated, and there are concerns about their accuracy and reliability during various conditions, such as surgery, hypoxia, and intravenous nutrition [1]. Therefore, bedside point-of-care capillary glucose testing (POC) is the standard of care to assess in-hospital glycaemic control and adjust insulin therapy.

Patients undergoing pancreatoduodenectomy (PD) have an increased risk of hyperglycaemia due to loss of beta-cell mass after PD, and this risk is further aggravated in postoperative management by parenteral nutrition therapy (TPN) [2–4]. There are concerns about TPN-induced hyperglycaemia, as the previous trials have shown increased length of hospital stay, increased risk of complications, and higher mortality in hospitalized patients [5, 6]. Therefore, early and aggressive intervention is recommended to prevent and correct hyperglycaemia in patients receiving TPN.

Consensus statement of the American Diabetes Association (ADA) defines hyperglycaemia in the hospital setting as any blood glucose levels greater than > 7.8 mmol/l (140 mg/dl) and recommend targets for glucose levels in hospitalized patients with premeal and random glucose targets of < 7.8 mmol/l (< 140 mg/dl) and < 10 mmol/l (< 180 mg/dl), respectively, for non-critically ill patients; and target ranges of 7.8–10 mmol/l (140–180 mg/dl) for critically ill patients [7, 8]. Intravenous insulin infusion is an effective treatment that quickly normalizes glucose. However, this treatment requires intensive point-of-care (POC) blood glucose monitoring, which is staff-intensive and, therefore, a limited option outside the intensive care unit (ICU) [9]. Continuous glucose monitoring (CGM) provides the advantage of measuring interstitial glucose every 5–15 min, providing a 24-h glycaemic profile. Recent studies comparing isCGM and POC capillary testing suggest that isCGM devices in the in-patient setting might help monitor non-critically ill patients on basal-bolus subcutaneous insulin regimen [10]. There are also few studies evaluating the accuracy of real-time CGM (rtCGM) in operating and ICU units [11, 12]. However, CGMs are developed for out-patient settings, and a recent consensus document evaluating the in-hospital use pointed out potential advantages but also barriers, concluding that CGMs cannot replace POC testing when monitoring blood glucose in the hospital [13].

To our knowledge, there are no studies evaluating the use of a hybrid approach using CGM system together with periodic POC testing during TPN-induced hyperglycaemia treated with continuous insulin infusion. The primary aim of this trial was to evaluate if a strict glucose intervention with insulin infusion monitored with a hybrid approach of isCGM and periodic POC is safe, practical, and beneficial in a surgical, non-ICU ward setting during TPN-induced hyperglycaemia. The secondary aim was to evaluate the accuracy of the isCGM system compared with POC testing.

## Methods

In January 2017, a novel regimen of strict and active control of blood glucose levels was introduced for all PD patients at Skåne University Hospital, Sweden. All adult patients, with or without diabetes, scheduled for elective PD at Skåne University Hospital in Lund, Sweden, between 2017 and 2019 were screened for participation before surgery. All study participants received oral and written information about the study and provided written informed consent for participation before any other study-related procedure was performed.

**Fig. 1:**
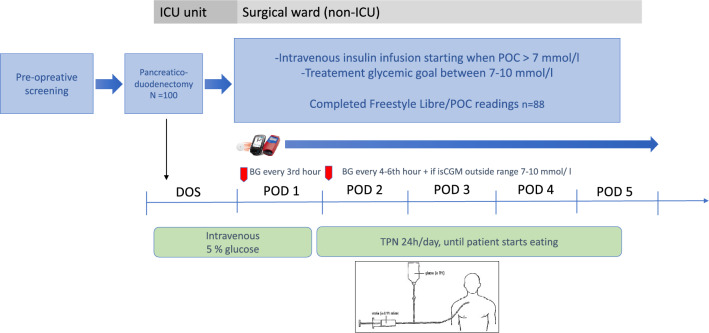
The postoperative protocol of isCGM/POC monitoring during day of surgery (DOS) and postoperative day (POD) 1-5

After surgery, all participants received a FreeStyle Libre 1 (Abbott Diabetes Care Inc., Alameda, CA, USA) isCGM sensor subcutaneously on the upper arm and were monitored with both isCGM and periodic POC testing, with a HemoCue® Glucose 201 DM system (HemoCue AB, Ängelholm, Sweden). POC glucose was monitored according to a protocol, at least every 3rd hour during the 1st postoperative day (POD1), see Fig. 1. From POD2, POC testing was performed at least every 4–6 h during the intravenous insulin regimen, and additionally, if the isCGM value was outside 7–10 mmol/l (125–180 mg/dl) to confirm the CGM value. isCGM was scanned between the obligate POC testing intervals to evaluate responses to insulin treatment. By routine, all patients received intravenous glucose 5% during the day of surgery (DOS) and POD1. From POD2, intravenous TPN was administered continuously for 24 h/day, until patients could start eating. All patients received continuous intravenous insulin infusion postoperative when POC glucose > 7 mmol/l (> 125 mg/dl), and insulin dose was titrated according to protocol with a sliding scale algorithm to maintain blood glucose between 7 and 10 mmol/l (125–180 mg/dl), to avoid risk of hypoglycaemia (Supplement, tables A and B). Insulin infusion was stopped, at least temporarily, when POC < 7.0 mmol/l (125 mg/dl), and definitely, when TPN was discontinued. The target glucose level of 7–10 mmol/l was adapted from ADA´s recommended target of 7.8–10.0 mmol/l during in-hospital insulin treatment, for most critically ill and non-critically ill patients, with a minor rounding of the lower glucose level to 7 mmol/l [8]. To evaluate a plausible change in glycaemic control, we compared all POC values from DOS to POD5 with POC values from a retrospectively recruited control group of PD patients from January 2015 until December 2016. Their hyperglycaemia was treated with multiple subcutaneous insulin injections (standard treatment) prescribed by a diabetologist on call and was monitored with POC only. To evaluate the safety of the novel intervention, every episode of hypoglycaemia (BG < 3.9 mmol/l, < 70 mg/dl) or severe hypoglycaemia (BG < 3.0 mmol/l, < 54 mg/dl) was reported. Further, all values where POC value differed more than 1 mmol compared with isCGM were manually analysed to evaluate safety.

The secondary aim was to calculate differences between matched POC and CGM values. Participants who met the analysis criteria of complete CGM and POC readings during 5 postoperative days were included in this analysis. Each POC value was time-matched with an isCGM value, and no correction for an eventual time lag was made. Absolute relative difference (%) between each POC and isCGM was calculated $\left(\sqrt{{\left(\frac{100\mathrm{x}\left(\mathrm{isCGM}-\mathrm{POC}\right)}{\mathrm{POC}}\right)}^{2}}\right).$

After testing for normal distribution, median absolute relative difference (MARD, %) with interquartile range (IQR) during 5 postoperative days was calculated. A Clarke error grid analysis was performed to evaluate the clinical significance of inaccuracies in the measurements of blood glucose concentration [14]. It compared the bias between the isCGM value and POC reference results. The data points were assigned to five zones (A–E) on the error grid. The results falling in zones A and B were considered clinically acceptable, meaning that the observed bias from laboratory results would not lead to treatment decisions that could put the patient at risk. Zone A represents glucose values that deviate from the reference by no more than 20%, or both CGM and POC values are in the hypoglycaemic range (< 3.9 mmol/l (< 70 mg/dl)). Upper and lower zone B represents values that deviate from the reference by > 20% but would lead to benign or no change in treatment. Zone C indicates than an over-corrective treatment might be given. Zone D represents values where an error is not detected, and thus, no corrective treatment is given. Zone E corresponds to values which could have dangerous consequences.

All analyses were performed using either Microsoft Excel (Microsoft, Redmond, WA, USA) or SPSS version 26 (IBM, NY, USA). Shapiro–Wilk´s test and visual inspection of histograms and Q-Q plots were performed to test data for normality [15]. Normally distributed data were expressed as mean and standard deviations (SD) and non-normally distributed as median with interquartile range (IQR). Categorical values are given as absolute numbers and the frequency in percentages, *n* (%). For continuous variable comparison, a *t*-test (if normally distributed data) and Mann–Whitney *U*-test (if non-normally distributed) were used. The Person chi2 test was used when analysing categorical variables, and the Fisher’s exact test was used if frequencies were less than 5. A two-tailed *p* < 0.05 was considered statistically significant.

This study was part of a larger trial aiming to evaluate the impact of an improved glucose control on postoperative complications after PD. The outcome in the complication trial is reported in a separate manuscript (Ekström et al. Hyperglycaemia and insulin infusion in pancreatoduodenectomy; feasibility and impact on complications. Submitted manuscript, 2023). The power calculation was mainly based to detect a difference in complication rates. At Skåne University Hospital, Sweden, approximately 45–55 pancreatoduodenectomies are performed yearly, with a postoperative morbidity rate of 65%. To detect a decrease in morbidity from 65 to 45%, with an *α*-value of 5% and a *β*-value of 80%, 75 patients in each group would be needed. Based on the power calculation, 100 patients were included in each group (intervention as well as historic control group).

## Results

During the study period from January 2017 to June 2019, 100 patients were included prospectively in the hybrid group (isCGM/POC), and 100 patients included retrospectively in the historical group. Clinical demographics of all patients are presented in Table 1.

**Table 1:** Patients clinical demographics. Values are numbers (percentages) except when median (interquartile range).

	Historical cohort *n* = 100	Intervention group *n* = 100	*p*-value
Median age (years)	68 (60–74)	70 (63–76)	0.139
Female gender, *n* (%)	38 (38)	47 (47)	0.198
Diabetes before surgery, *n* (%)	21 (23)	23 (23)	0.733
Preoperative insulin treatment, *n* (%)	13 (13)	15 (15)	0.684
Insulin treatment at discharge from hospital, *n* (%)	18 (18)	21 (21)	0.722
Heart disease, *n* (%)	12 (12)	21 (21)	0.086
Median eGFR < 60 ml/min/1.73 m^2^	81 (63–98)	85 (75–99)	*0.042*
Baseline CRP (mg/l)	3.8 (1.5–10.8)	3.9 (1.9–10.5)	0.620
Max CRP (mg/l) during POD1-5	183 (109–267)	160 (115–236)	0.124

*p-value <0.05* is considered statistical significant

**Fig. 2:**
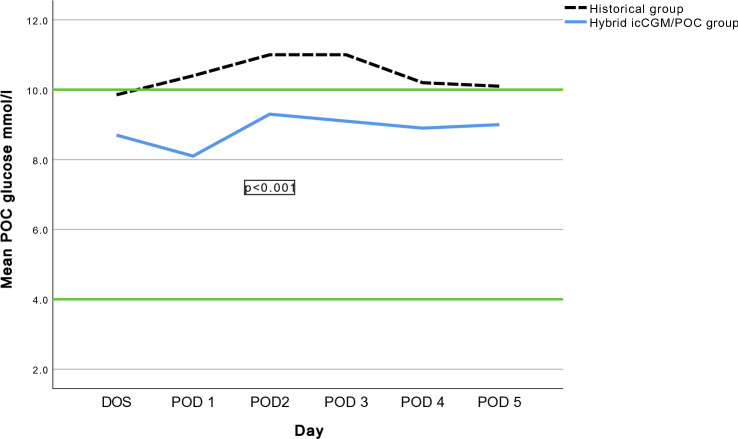
Mean daily POC glucose in the intervention group and in the historical control, during day of surgery (DOS) and postoperative day (POD) 1-5. *p* < 0.001. Blue solid line = Mean daily POC in intervention group receiving insulin infusion with hybrid isCGM/POC monitoring. Black dotted line = Mean daily POC in historical control group receiving standard treatment with multiple subcutaneously insulin injections

The hybrid approach (isCGM/POC) and insulin infusion significantly lowered mean glucose compared with the historical control group, Fig. 2. Mean glucose during DOS-POD5 was 8.8 ± 2.2 mmol/l (158 ± 40 mg/dl) in the hybrid group, compared with 10.4 ± 3.4 mmol/l (187 ± 61 mg/dl) (*p* < 0.001) in the control group.

In the isCGM/POC group, 88 patients had successful isCGM readings during all five POD, and matched POC and CGM values were evaluated for MARD. Twelve patients were excluded from the MARD analysis due to sensor loss, accidental removal of sensor, sensor or reader dysfunction or sensor reader loss.

**Fig. 3:**
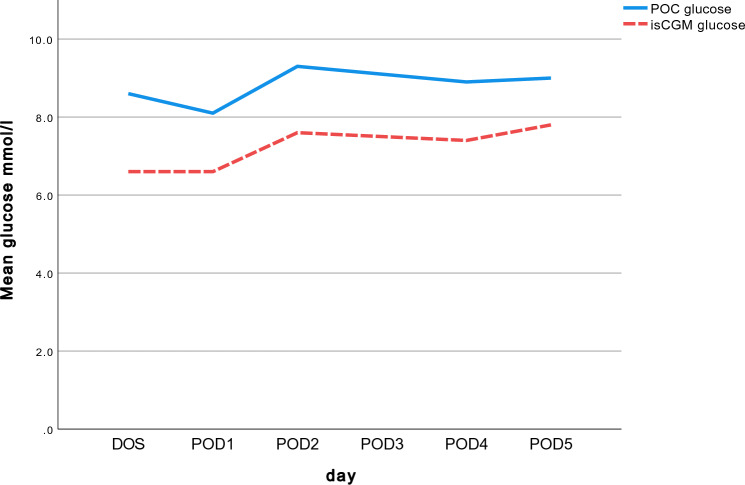
Mean daily POC glucose and isCGM glucose during day of surgery (DOS) and postoperative day (POD) 1-5 in the intervention group. *p* < 0.001. Blue solid line = POC capillary testing. Red dotted line = Freestyle Libre isCGM

During POD1-5, a total number of 2346 paired POC-CGM were evaluated. Mean POC glucose was higher (8.4 ± 2.1 mmol/l) than mean isCGM readings (7.0 ± 2.2 mmol/l, *p* < 0.001), as illustrated in Fig. 3, and isCGM was lower than POC values in 91% of all readings.

**Fig. 4:**
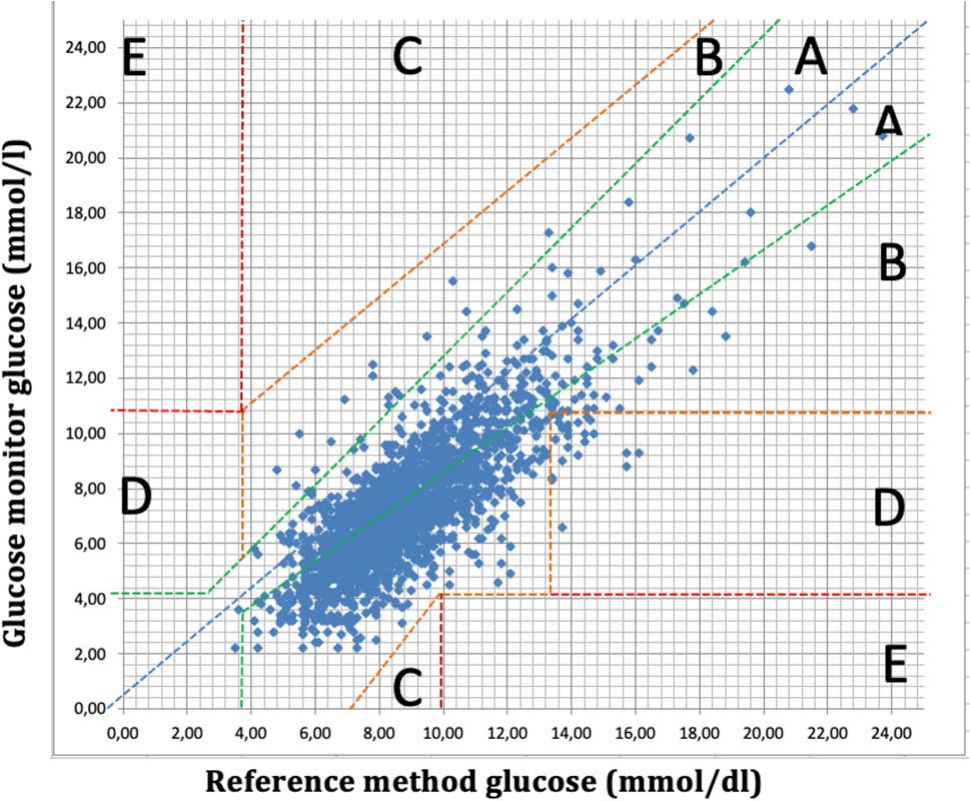
Clarke error grid analysis. In this data set, 57.2% of values fell in zone A, 41.8% in zone B, 0,4% in zone C, 0.6% in zone D, and none in zone E

The majority (77.3%) of POC glucose measurements were within the range 4.0–10.0 mmol/l (72–180 mg/dl), 22.6% were above 10 mmol/l, and 0.1% were below 4.0 mmol/l (*n* = 6), with a minimum value of 3.5 mmol/l. Corresponding numbers for isCGM were 84.9%, 10.5%, and 4.6%, respectively. Of all CGM readings, 4.5% were < 3.9 mmol/l (70 mg/dl), but only six of these events were confirmed with POC testing, and none was < 3.0 mmol/l (54 mg/dl). The Clarke error grid analysis showed that 99% of all paired POC-isCGM values were within zones A and B, see Fig. 4. Of all readings, only 0.4% were in zone C, 0,6% were in zone D, and no values were in zone E.

Of all the 2346 paired isCGM/POC readings, only 9% resulted in a lower POC value compared with the isCGM result. Of them, 47 differed between − 1 and − 2 mmol/l, and in 30 readings, the POC value was more than 2 mmol/l lower than the corresponding isCGM value.

Overall, MARD was 17.8% (IQR 10.2–26.7). Accuracy was lower within the lower glucose range (for glucose < 5.0 mmol/l), MARD was 28.6% (IQR 14.0–34.7). Further, MARD was highest during DOS (21.8%) and improved during the following days (MARD POD1 19.2%, POD2 18.5%, POD3 19.2%, POD4 18.1%, and POD5 14.2%).

The postoperative inflammatory stress, measured as CRP, was similar between groups, as shown in Table 1.

## Discussion

This is, to our knowledge, the largest in-hospital study evaluating a hybrid approach with isCGM and intermittent POC glucose to monitor and manage intravenous insulin infusion in a non-ICU setting after PD. Although isCGM readings generally are lower than simultaneous POC measurements, this study suggests isCGM be a safe method for glucose monitoring also in this setting and that the number of accompanying POC measurements could be fewer than in the present study protocol. This hybrid approach was also generally appreciated by the surgical ward nurses.

It is a well-known problem with hyperglycaemia during parenteral nutrition [5, 16], and among patients undergoing PD, this risk is even higher due to the loss of beta-cell mass after surgery [3, 4, 17]. Traditionally, most of these patients are given subcutaneous bolus insulin to treat hyperglycaemia, often with a non-optimal glycaemic control during the 1st postoperative days. With intravenous insulin, it is possible to reach glycaemic target faster, but this regimen traditionally requires intensive monitoring and is considered staff expensive, which limits its use on outside ICU units. Our study demonstrates that it is possible to manage this insulin regimen in a surgical, non-ICU ward using a hybrid glucose monitoring combining isCGM and POC measurements.

With 2346 matched POC-CGM pairs analysed, the study is also the largest in-hospital comparison between isCGM and POC glucose. We can conclude that isCGM values significantly underestimated glucose, with an overall MARD of 17.8%. The lowest accuracy was found within the lower glucose readings, and during the 1st days, with improving values throughout the period.

The previous studies of Freestyle Libre 1 in real-world out-patient settings have demonstrated MARD between 11 and 14% [18–21]. Also, in these studies, FreeStyle Libre reported lower glucose values compared with capillary testing. There are few studies evaluating FreeStyle Libre in an in-hospital setting, and our trial is the first to evaluate FreeStyle Libre isCGM during TPN and insulin infusion in elective and stable postoperative patients outside the ICU in a surgical ward. In a trial by Schierenbeck et al., evaluating FreeStyle Libre isCGM in 26 patients in an ICU setting during cardiac surgery, a MARD of 28.8% was demonstrated compared with arterial blood glucose, which is higher than our result [22]. Another ICU trial by Ancona et al., evaluating FreeStyle Libre in eight patients, demonstrated a MARD of 14%, compared with arterial blood glucose. The MARD was higher when compared with capillary testing [23]. In a study by Galindo et al., FreeStyle Libre isCGM was evaluated among 97 hospitalized patients with type 2 diabetes treated with basal-bolus insulin regimen in a non-ICU setting. In that study, the authors demonstrated that FreeStyle Libre values were significantly lower compared with POC values, with an overall MARD of 14.4% among 1829 matched POC-CGM glucose pairs [10].

One could speculate why FreeStyle Libre seems to have lower accuracy (higher MARD) during in-hospital use. The FreeStyle Libre isCGM measures interstitial glucose every 15 min. Glucose is transported from the capillaries into the interstitial fluid through passive diffusion. There is a time lag between changes in plasma and interstitial glucose levels. Software programmes and algorithms have been designed to accommodate the lag in glucose readings from the interstitial fluids. Despite this, rapid changes in blood glucose have been shown to affect the magnitude of differences more [21, 24]. This could have impacted our result since both intravenous nutrition and intravenous insulin, instantly affect glucose levels. Further, impaired microcirculation during critical illness, with altered body temperature, hypoperfusion, and hypoxia, has been shown to lower capillary glucose levels, making them more consistent with venous [25, 26]. These factors may considerably impact interstitial glucose levels in the upper arm, affecting isCGM accuracy.

Our results showed that the system in a postsurgical setting with stable patients was safe concerning hypoglycaemic events. Only 4.5% of all CGM readings were < 3.9 mmol/l (70 mg/dl) and of them only six events were confirmed with POC testing, and none was severe (< 3.0 mmol/l). The Clark error grid analysis showed that 99% of all our values were within zones A and B, meaning that the difference in value between the Freestyle Libre and the POC value was clinically insignificant. This result complies with the recommendations that at least 99% of measurement results shall fall within zones A and B of the grid [27].

However, we can conclude that isCGM almost consequently underestimated glucose, and a MARD of 17.8% indicates that values must be interpreted with caution in clinical practice when dosing insulin.

As CRP elevation was similar between groups, it is not likely that differences in postoperative inflammatory stress or infection explain the improvement in metabolic control in the isCGM group.

One limitation in our trial is, of course, the use of the first-generation FreeStyle Libre system on the market, as our trial was conducted 2017–2019. Today, technologically improved sensors and algorithms have been developed, which might increase the system´s accuracy and feasibility in an in-hospital setting. Another limitation is the use of a historic control group to assess improvement of metabolic control in our intervention group. Although the glucose lowering goal in the postoperative period was similar, other factors than differences in insulin administration method and glucose monitoring might have influenced outcome.

One strength of this study is the large number of patients and matched glucose pairs. Another strength is the real-world design in the surgical ward, with ordinary medical staff (i.e. nurses) managing the system during postoperative care in a non-ICU setting. Further, the study was performed entirely independently of the FreeStyle Libre manufacturer.

In summary, we can conclude that a hybrid approach with FreeStyle Libre isCGM/POC to monitor and manage intravenous insulin infusion in a non-ICU setting after PD is a safe and effective treatment option in a non-ICU setting after pancreatoduodenectomy. Although the isCGM system generally underestimates capillary glucose, using a hybrid approach, the CGM system increases the number of measuring points and could reduce the frequency of capillary testing, thus reducing the workload among the medical staff during insulin infusion.

### Supplementary Information

Below is the link to the electronic supplementary material.

Supplementary file1 (DOCX 17 kb)

## Abbreviations

PD: Pancreatoduodenectomy
ADA: American Diabetes Association
isCGM: Intermittently scanned continuous glucose monitor
POC: Point of care
TPN: Total parenteral nutrition
DOS: Day of surgery
POD: Postoperative day
BG: Blood glucose
MARD: Median absolute relative difference
SD: Standard deviations
IQR: Interquartile range
ICU: Intensive care unit
