# Supplementary material for: Intermittent scanning continuous glucose monitoring is safe and useful in postsurgical glucose monitoring after pancreatoduodenectomy
Source: Acta Diabetol. 2023 Aug 4;60(12):1727–33. doi: 10.1007/s00592-023-02158-0 (PMC10587023; doi:10.1007/s00592-023-02158-0)
Supplement: Supplementary file 1 — Supplementary file1 (DOCX 17 kb) [file 592_2023_2158_MOESM1_ESM.docx]

**Supplement:**

1. **Insulin start-up protocol**

| **Blood glucose (confirmed with POC)** | **Insulin dosage at start (1E/ml)** |
| --- | --- |
| <7.0 mmol/l | 0 ml/h |
| 7.0 - 8.0 mmol/l | 0.5 ml/h |
| 8.1 - 10.0 mmol/l | 1 ml/h |
| 10.1 - 13.0 mmol/l | 2 ml/h |
| 13.1 - 16.0 mmol/l | 3.5 ml/h |
| 16.1 - 20.0 mmol/l | 5 ml/h |
| >20 mmol/l | - 1. ml/h |

1. **Protocol for adjustment of insulin dosage**

| **Glucose value** | **Adjustment of insulin dosage** |
| --- | --- |
| If glucose is < 5 mmol/l  **(Confirmed with POC-testing)** | Stop the infusion and give 10 ml Glucose 300 mg/ml iv. Do another POC-testing after 0.5 hours. If blood glucose is ≥7 mmol/l the infusion is restarted with half the previous speed. |
| If glucose is 5.0-6.9 mmol/l  **(Confirmed with POC-testing)** | Stop the infusion. Do another testing after 0.5 hours. If POC glucose is then ≥7 mmol/l the infusion is restarted with half the previous speed. |
| If glucose 7.0-10.0 mmol/l and: (a.b or c. below)   1. is stable (at least 2 readings within range) 2. has declined with > 50% compared to previous value. 3. has declined with < 50% compared with previous value, but is close to 7.0 mmol/l. | 1. Unchanged dosage. isCGM scanning after 1 hour and if still stable between 7.0-10-0 mmol/l continue scanning every 2^nd^ hour, and POC testing every 3^rd^ to 6^th^ hour. 2. Reduce the speed by half. POC-testing after 0.5 hour. 3. Reduce the dosage by 25%. POC testing after one hour. |
| If blood glucose is >10 mmol/l  (Confirmed with POC testing) | Increase the speed by 1 ml/hour, unless the POC value has declined compared with previous value and is getting close to range (then keep unchanged dose). isCGM scanning after 1 hour. |
